# Compressive Properties and Energy Absorption Characteristics of Co-Continuous Interlocking PDMS/PLA Lattice Composites

**DOI:** 10.3390/ma17163894

**Published:** 2024-08-06

**Authors:** Han Wang, Kedi Wang, Jincheng Lei, Xueling Fan

**Affiliations:** 1 Xi’an Key Laboratory of Extreme Environment and Protection Technology, School of Aerospace Engineering, Xi’an Jiaotong University, Xi’an 710049, China; 2 International Center for Applied Mechanics, State Key Laboratory for Strength and Vibration of Mechanical Structures, Xi’an Jiaotong University, Xi’an 710049, China

**Keywords:** compressive properties, energy absorption characteristics, lattice structure, co-continuous composites, interlocking structure

## Abstract

Co-continuous interlocking lattice structures usually present superior compressive properties and energy absorption characteristics. In this study, co-continuous interlocking polydimethylsiloxane/polylactic acid (PDMS/PLA) lattice composites were designed with different strut diameters, and successfully manufactured by combining the fused deposition modeling (FDM) technique and the infiltration method. This fabrication method can realize the change and control of structure parameters. The effects of the strut diameter on the compressive properties and energy absorption behavior of PDMS/PLA lattice composites were investigated by using quasi-static compression tests. The compressive properties of the co-continuous interlocking PDMS/PLA lattice composites can be adjusted in a narrow density range by a linear correlation. The energy absorption density of the co-continuous interlocking PDMS/PLA lattice composites increases with the increase in the PLA strut diameter and presents a higher efficiency peak and wider plateau region. The PLA lattice acts as a skeleton and plays an important role in bearing the compressive load and in energy absorption. The indexes of the compressive properties/energy absorption characteristics and PLA volume fraction of co-continuous interlocking PDMS/PLA lattice composites show linear relationships in logarithmic coordinates. The effect of the PLA volume fraction increasing on the plateau stress is more sensitive than the compressive strength and energy absorption density.

## 1. Introduction

The materials and structures observed in nature often showcase many captivating architectural designs and exceptional characteristics [1,2,3]. Classical porous materials, including honeycomb structures and foam structures, show unique properties such as low density and high specific strength and stiffness [4,5,6]. Inspired by nature, porous structures find applications in vehicles, aircrafts, biological scaffolds and other fields [7,8]. Besides superior mechanical properties, special functional properties such as energy absorption, sound absorption, electromagnetic shielding, damping, permeability and biocompatibility render porous materials highly promising for practical applications [9,10,11,12,13].

Currently, the investigations of lattice structures have received extensive attention [14,15,16,17]. In comparison to foam structures with stochastic cells, lattice structures offer the advantages of optimal design and precise control, enabling them to meet the requirements for the special and adjustable properties [18]. This is precisely why lattice structures have garnered significant attention from researchers. Additionally, the rapid advancement of additive manufacturing has led to the widespread use of lattice structures as one of the most commonly employed porous architectures. Nonetheless, the design of a lattice structure alone cannot meet the higher requirements of energy absorption targets like vehicle collision and armor protection. There is a view that introducing the second solid phase, instead of the gas phase of a lattice structure, is believed to offer a combination of the advantages of the filler material and the original lattice skeleton, leading to a significant improvement in mechanical properties [19,20,21,22]. Hence, the co-continuous composites achieved through this method [23,24,25,26] are proposed, and the enhancement objective of mechanical properties is successfully achieved.

The co-continuous composites typically consist of two distinct materials in order to allow for the adjustment of the global properties of co-continuous composites. The filler materials must satisfy specific conditions in terms of preparation method feasibility and enhancement of mechanical properties when compared to the original lattice skeleton [21]. Therefore, the two phases of co-continuous composites are usually chosen from metals, ceramics, polymers and even liquid phase materials. Wang et al. [23] demonstrated the potential to design and fabricate co-continuous glassy polymer/rubbery polymer materials with enhancements in stiffness, strength and energy dissipation using a 3D printer. Three types of co-continuous structures with simple cubic (SC), body-centered cubic (BCC) and face-centered cubic (FCC) Bravais lattices were considered in their study. Liu et al. [24] investigated the quasi-static compressive behaviors of the co-continuous glassy polymer/liquid composites. Due to the presence of liquid filler, the stiffness, yield strength and energy absorption of co-continuous composites were significantly enhanced, which comes from the additional support of the liquid filler and the lateral expansion of the glassy polymer. Geometric structures of the glassy polymer, namely a simple cubic (SC) lattice, a face-centered cubic (FCC) lattice and a body-centered-cubic (BCC) lattice, were investigated. Al-Ketan et al. [27,28] employed 3D printing technology to fabricate interpenetrating phase composites (IPCs) and investigated the mechanical properties of IPCs with different periodic architectures comprising a soft matrix and a continuous, smooth-curved, hard material. Additionally, Al-Ketan et al. [25] proposed a nature-inspired 3D periodic shell–core cellular co-continuous composites made of a hard-shell and soft-core system based on a Gyroidal triply periodic minimal surface. Mansouri et al. [29] investigated the mechanical behavior of a 3D periodic single-material cellular D-structure and the corresponding co-continuous composite combining hard thermoplastic materials with soft rubbers with a rupture strain of more than 500%, fabricated using multimaterial fused deposition modeling (FDM) technology. Wang et al. [21] prepared Mg_17_Al_12_/Al ordered structure composites by infiltrating the ordered porous aluminum with the intermetallic compound Mg_17_Al_12_ under gravity conditions. The intermetallic compound Mg_17_Al_12_ exhibits significant brittleness while the ordered porous aluminum shows excellent plasticity and ductility. Chen et al. [30] propose to introduce two-phase co-continuous composites as acoustic metamaterials. The two phases of co-continuous composites were chosen to be a ceramic (boron carbide) and a glassy polymer (epoxy). The aforementioned analysis indicates that co-continuous composites have the potential to enhance mechanical properties and optimize functional properties, regardless of the chosen preparation method. The changes in the deformation mechanism and failure mode caused by the presence of the co-continuous interpenetrating phase should be taken into consideration, and the effect of co-continuous interpenetrating structure parameters on mechanical properties should be investigated.

In this study, polylactic acid (PLA) lattices with simple cubic structure were fabricated using the FDM technique as an infiltration skeleton and then filled with liquid polydimethylsiloxane (PDMS) under gravity conditions. The co-continuous interlocking PDMS/PLA lattice composites were prepared following a combination of the FDM technique and the infiltration method. The effect of the strut diameter on the compressive properties and energy absorption behavior of the PDMS/PLA lattice composites were investigated using quasi-static compression tests. The cooperative deformation mechanism and failure mode of the co-continuous interlocking PDMS filler and PLA matrix skeleton were analyzed. The objective of this study was to demonstrate that modifying the parameters of the co-continuous interlocking structure could effectively enhance compressive properties and energy absorption characteristics.

## 2. Materials and Methods

### 2.1. Design of Co-Continuous Interlocking PDMS/PLA Lattice Composites

**Figure 1:**
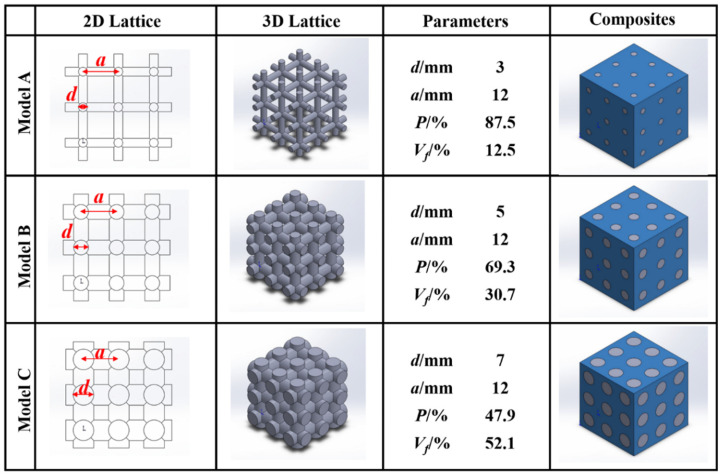
Illustration of 2D/3D PLA lattice structures, corresponding structure parameters and 3D co-continuous interlocking PDMS/PLA lattice composites.

The co-continuous interlocking lattice structures in this study were designed and modeled using the software SOLIDWORKS 2018. The PLA lattice structures consisted of several orthogonal cylinders with different strut diameters based on previous studies [16]. Figure 1 shows the 2D/3D PLA lattice structures, the 3D co-continuous interlocking PDMS/PLA lattice composites and their corresponding structure parameters including the strut diameter *a* (3, 5, 7 mm), the strut spacing *d* (12 mm), the porosity *P* and the volume fraction *V_f_*. The overall dimensions of the CAD models are 36 mm × 36 mm × 36 mm. The volume fractions *V_f_* of the PLA lattice A, B and C are 12.5%, 30.7% and 52.1%, respectively. The corresponding porosities are 87.5%, 69.3% and 47.9%, namely the volume fractions of the PDMS. With the strut diameter increasing, the volume fractions *V_f_* of the PLA increase and the volume fractions of the PDMS decrease. 

### 2.2. Preparation of Co-Continuous Interlocking PDMS/PLA Lattice Composites

The PLA lattice structures were prepared using a 3D printer (Creator-pro, Hangzhou, China) with the FDM technique [31,32] in this investigation. The raw material of the 3D printer is a commercial wire PLA product (FLASHFORGE, Hangzhou, China). The models of the PLA lattice structures were sliced and imported into the 3D printer. The corresponding 3D printing parameters are as follows: slice thickness, 0.12 mm, nozzle preheating temperature, 200 °C, printing speed, 50 mm/s, printing accuracy, ±0.4 mm, etc. After the 3D printing process, the extra support materials were removed and the PLA lattice structures were cleaned manually. 

**Figure 2:**
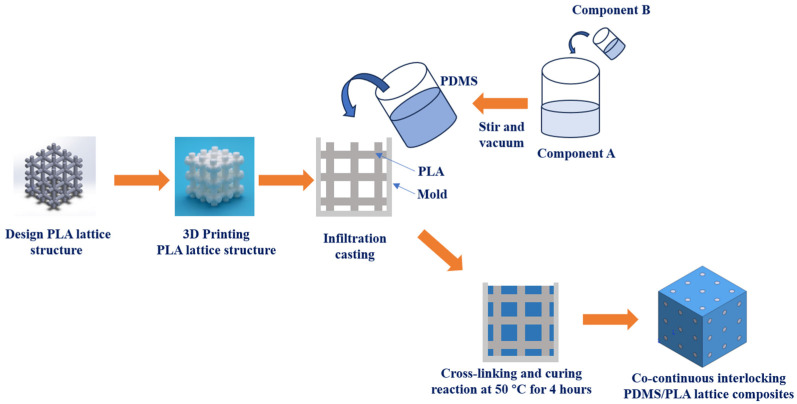
The fabrication process of co-continuous interlocking PDMS/PLA lattice composites.

Figure 2 shows the fabrication process of the co-continuous interlocking PDMS/PLA lattice composites. The 3D-printed PLA lattice structure was set as an infiltration skeleton and placed in the corresponding infiltration mold (FLASHFORGE, Hangzhou, China). The PLA lattice structure was in contact with the four internal surfaces of the mold and surrounded by the mold. The PDMS (Sylgard 184, Tianjin, China) was prepared by mixing component A (monomers, Sylgard 184, Tianjin, China) and component B (crosslinkers, Sylgard 184, Tianjin, China) with the mass ratio of A:B = 10:1. Component A was poured into a beaker and then component B was dripped into component A. The mixture was stirred for 15 minutes and vacuumed to remove air bubbles from the solution. The PDMS was then utilized to infiltrate the PLA lattice structure in the infiltration mold under a slight vibration at room temperature. After the PDMS infiltration process, the PDMS/PLA mixture was placed into a vacuum furnace (SHKTYQ, Shanghai, China) and heated at 50 °C for 4 hours. At this temperature, the PDMS cross-linking and curing reaction occurred. Eventually, the external mold was removed and the specimens of co-continuous interlocking PDMS/PLA lattice composites were successfully prepared. The fabrication process of the co-continuous interlocking PDMS/PLA lattice composites consists of two main stages, 3D printing and infiltration casting. As for limitations in 3D printing, there may be printing size errors or anisotropy. As for limitation in infiltration casting, the infiltration of filler may introduce infiltration defects, such as a small number of bubbles.

### 2.3. Characterization of Co-Continuous Interlocking PDMS/PLA Lattice Composites

All specimens of the PLA lattice structures and co-continuous interlocking PDMS/PLA lattice composites were photographed with a digital camera (Nikon, S6300, Tokyo, Japan) and weighed with an analytical electronic balance. The densities of specimens were calculated according to the mass and structure parameters. The quasi-static compression tests were performed on an electromechanical universal mechanical testing machine (MTS, C45.105, Eden Prairie, MN, USA) at an initial strain rate of 10^−3^ s^−1^ to evaluate the compressive properties and energy absorption behavior of the co-continuous interlocking PDMS/PLA lattice composites. To ensure data stability and repeatability, at least three compressive specimens were tested for each condition at room temperature. The compressive strength, plateau stress, densification strain, energy absorption density and energy absorption efficiency were calculated. Macro images of specimens at different compressive strains were recorded during the quasi-static compression tests. The deformation and failure processes of the co-continuous interlocking PDMS/PLA lattice composites were analyzed.

## 3. Results and Discussion

### 3.1. Structure Characterization of Co-Continuous Interlocking PDMS/PLA Lattice Composites

**Figure 3:**
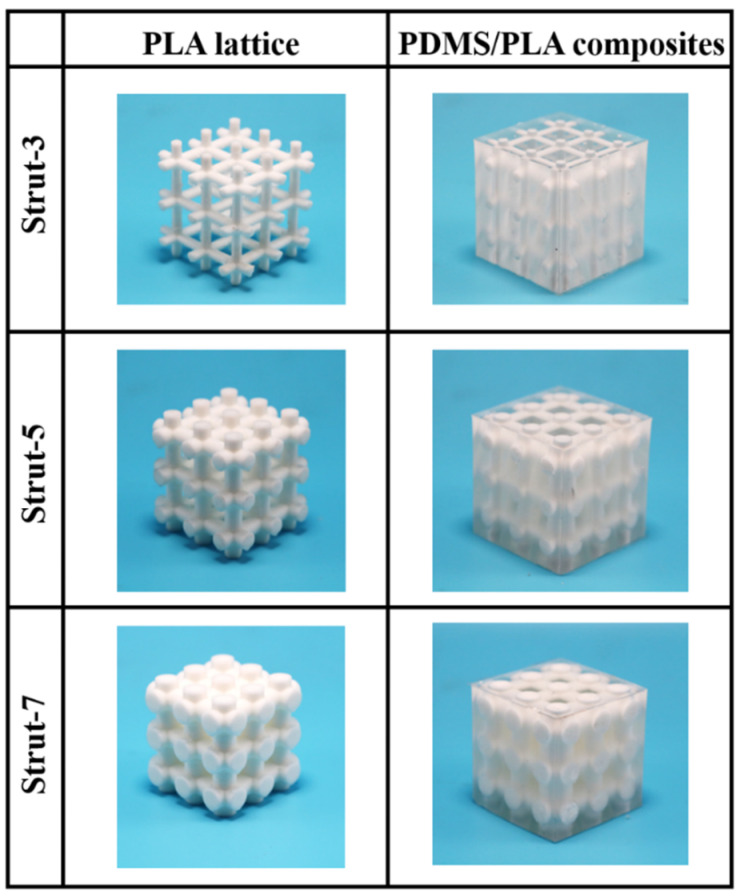
The specimens of PLA lattice structures and co-continuous interlocking PDMS/PLA lattice composites.

The specimens of the PLA lattice structures and co-continuous interlocking PDMS/PLA lattice composites are shown in Figure 3. The PLA lattice exhibits a basic truss structure with different strut diameters of 3, 5 and 7 mm. It is observed that the specimens of the co-continuous interlocking PDMS/PLA lattice composites consist of two parts, namely the PLA lattice structure as a skeleton and the PDMS as a filler. These two components become an integral structure with co-continuous interpenetrating phases. The co-continuous interpenetrating structure characteristic of the PDMS and PLA can be visually seen from the specimens’ appearance due to the transparent features of the PDMS. The changes in strut diameter and the infiltration treatment cause variations of specimen mass and density, which should be considered when evaluating mechanical properties and energy absorption characteristics. The specimen label, filler, strut diameter, mass and density are shown in Table 1. Compared with the specimens made by Al-Ketan et al [25,27,28], Wang et al. [26] and Mansouri et al. [29], the specimens in this study avoided the filler anisotropy caused by 3D printing. Compared with the specimens made by Wang et al. [21], the specimens in this study show fewer defects due to the longer infiltration time. 

### 3.2. Compressive Properties of Co-Continuous Interlocking PDMS/PLA Lattice Composites

**Figure 4:**
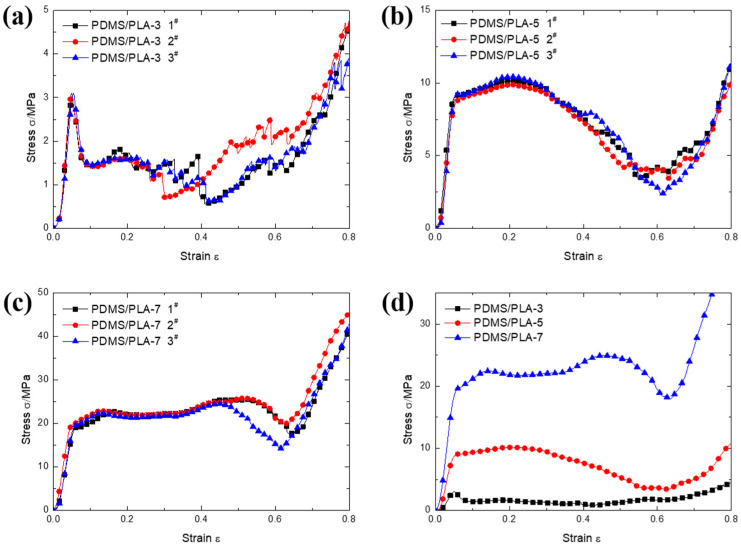
The compressive stress–strain curves of co-continuous interlocking PDMS/PLA lattice composites (**a**) PDMS/PLA-3, (**b**) PDMS/PLA-5, (**c**) PDMS/PLA-7 and (**d**) the comparison among them.

The compressive stress–strain curves of co-continuous interlocking PDMS/PLA lattice composites PDMS/PLA-3, PDMS/PLA-5 and PDMS/PLA-7 are shown in Figure 4. For each type of specimen, three compressive stress–strain curves are plotted here. For the PDMS/PLA-3 specimens, a prominent linear elastic stage appears initially followed by an obvious stress peak. The stress declines quickly after reaching its peak, indicating the occurrence of a stress drop. Subsequently, the stress enters a second stage, namely the plateau region. It is found that the stress in this region fluctuates. Finally, the stress increases rapidly again and enters the densification stage. The compressive stress–strain curves of the PDMS/PLA-5 specimens show different characteristics. First, the stress levels of the PDMS/PLA-5 specimens are obviously higher than those of the PDMS/PLA-3 specimens because of the larger effective cross-section area to bear load. Second, there is no stress drop after the linear elastic stage. Instead, the stress enters a plateau region directly. This indicates that the PDMS/PLA-3 specimens show buckling deformation behavior because of the relatively slenderer strut, while the PDMS/PLA-5 specimens show compressive deformation behavior. Therefore, the plateau stress of the PDMS/PLA-5 specimens is approximately close to the compressive strength. After the plateau region, the stress decreases slowly to the minimum value. Eventually, the stress increases quickly again and the densification stage begins. For the PDMS/PLA-7 specimens, the compressive stress–strain curves still comprise three stages with typical characteristics. The stress level of the whole compression process is further increased compared with the PDMS/PLA-3 specimens and PDMS/PLA-5. For the PDMS/PLA-7 specimens, there is no sudden stress drop like for the PDMS/PLA-3 specimens. The stress is maintained near the plateau stress and then decreases slowly to a stress valley at around strain 0.6. It is worth noting that the width of the plateau region with high and stable stress is wider than that of the PDMS/PLA-3 specimens and PDMS/PLA-5. At the same time, the stress fluctuations are relatively more moderate. The strut diameters of PDMS/PLA-7 are larger and the specimens exhibit a stronger capacity of bearing loads. Figure 4d shows the comparison of stress–strain curves among the co-continuous interlocking PDMS/PLA lattice composites PDMS/PLA-3, PDMS/PLA-5 and PDMS/PLA-7. Each curve was acquired by averaging by the three repeated experiments. It can be directly seen that the stress level is improved significantly. The compressive strength and the plateau stress increase with the strut diameter increasing from 3 to 7 mm.

**Figure 5:**
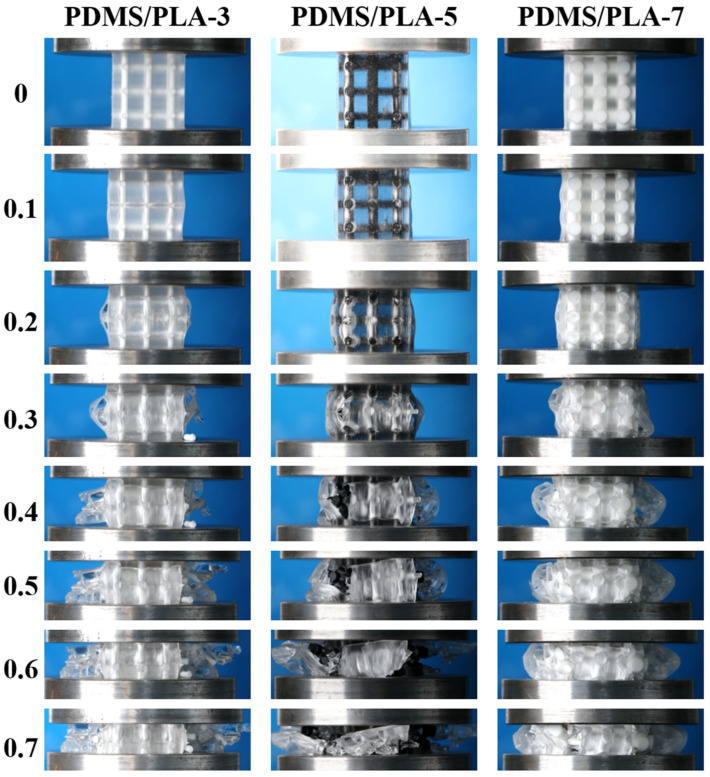
The compressive stress–strain curves of co-continuous interlocking PDMS/PLA lattice composites PDMS/PLA-3, PDMS/PLA-5, and PDMS/PLA-7.

The quasi-static axial compression tests of the co-continuous interlocking PDMS/PLA lattice composites PDMS/PLA-3, PDMS/PLA-5 and PDMS/PLA-7 at different compressive strains (0, 0.1, 0.2, 0.3, 0.4, 0.5, 0.6 and 0.7) are shown in Figure 5. According to Maxwell’s criterion, the dominated mechanism of simple cubic structure is the stretching-dominated mechanism. For the PDMS/PLA-3 specimens, the PLA lattice as the skeleton played a main role in bearing the compressive load due to the higher Young’s modulus. At the initial stage of compression deformation, the PLA lattice showed linear elastic deformation. With the compressive load increasing, the struts buckled, resulting from the high ratio of length to diameter. At the plateau region, the PLA struts were compressed and bended. Even the joints cracked due to the large stress concentration. Eventually, the PLA struts ruptured and peeled from the specimen. The PLA lattice collapsed layer by layer and entered the densification stage. During the whole compression process, the PDMS acted as a flexible material and wrapped the PLA strut. The compression and lateral expansion of the PDMS filler occurred, hindering the deformation of the PLA. This made a difference in the deformation process of the PLA lattice. The exitance of the PDMS as a filler slows the tendency of the PLA strut to deform and fracture, reflecting that the co-continuous interlocking structure contributes to the improvement of global mechanical properties.

Compared with the compression process of the PDMS/PLA-3 specimen in Figure 5, the compression deformation processes of the PDMS/PLA-5 specimens and PDMS/PLA-7 are similar. Initially, the whole specimen deformed linearly. Subsequently, under the influence of the compression load, the PLA lattice ruptured and the PDMS in the middle of the specimen was deformed by expansion at the plateau region. Finally, the specimen entered the densification stage and more PDMS was expelled from the PLA lattice. It is worth noting that the strut diameters of the PDMS/PLA-5 specimens and PDMS/PLA-7 are larger than that of specimen PDMS/PLA-3. Therefore, the PLA lattice played a more important role in bearing compressive loads. Similarly, the stretching-dominated mechanism was the main deformation mechanism. The struts wrapped by PDMS filler exhibited more superior mechanical properties and stronger stability. This characteristic proves the advantages of a co-continuous interlocking structure. Meanwhile, increasing the strut diameter of the PLA skeleton is beneficial to improve the mechanical properties.

**Figure 6:**
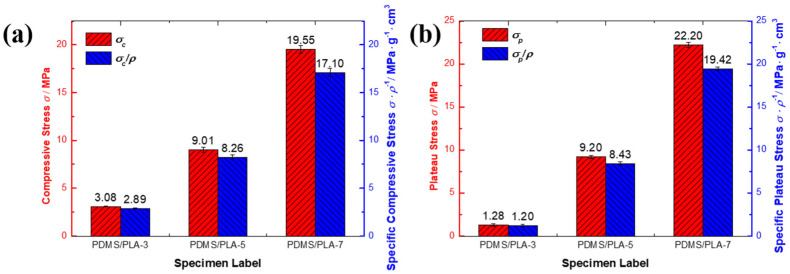
The compressive property indexes: (**a**) compressive strength; (**b**) plateau stress of co-continuous interlocking PDMS/PLA lattice composites.

The compressive property indexes of the co-continuous interlocking PDMS/PLA lattice composites are shown in Figure 6. The compressive property indexes include the compressive strength *σ_c_*, the specific compressive strength *σ_c_*/*ρ*, the plateau stress *σ_p_* and the specific plateau stress *σ_p_*/*ρ*. The compressive strength is usually taken as the first stress peak of the compressive stress–strain curve. If there no local maximum occurs, the compressive strength is determined at the 1% strain offset. The plateau stress is usually calculated by averaging the stress from strain 0.2 to strain 0.4. For specimen PDMS/PLA-3, the compressive strength was 3.08 MPa and the plateau stress was 1.28 MPa. It was found that the plateau stress was 41.56% of the compressive strength. The lowest plateau stress is not conducive to bearing loads and absorbing energy. The PLA lattice with 3 mm struts as the skeleton of the co-continuous interlocking structure showed poor load-carrying capacity. Although there is more PDMS as filler, the strut diameter of specimen PDMS/PLA-3 is too small. Therefore, the global mechanical properties are inferior. The PDMS/PLA-5 specimen showed higher compressive strength (9.01 MPa) and plateau stress (9.20 MPa). With the increase in strut diameter (5 mm), the compressive strength and the plateau stress increased around 1.93 times and 6.19 times compared with those of the PDMS/PLA-3 specimen, respectively. It was found that the compressive strength and the plateau stress are almost identical. This result is beneficial to bearing load and absorbing energy. The PDMS/PLA-7 specimen exhibited the highest compressive strength (19.55 MPa) and plateau stress (22.20 MPa) in this study. Though the PDMS/PLA-7 specimen has a similar structure to PDMS/PLA-3 and PDMS/PLA-5, it has the largest strut diameter (7 mm) for the PLA lattice. The compressive strength and the plateau stress were 2.17 times and 2.41 times of the PDMS/PLA-5 specimen, respectively. Meanwhile, the plateau stress was close to the compressive strength of specimen PDMS/PLA-5. Considering the wider plateau region, the PDMS/PLA-7 specimen showed a superior capacity of bearing load and absorbing energy.

**Figure 7:**
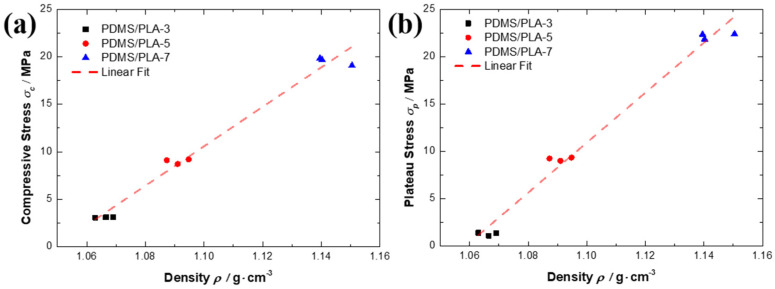
The compressive strength (**a**)/plateau stress (**b**) and corresponding density *ρ* of the co-continuous interlocking PDMS/PLA lattice composites.

The increase in strut diameter affects the density of the co-continuous interlocking PDMS/PLA. Considering the effect of the density, the specific compressive strength and the specific plateau stress of the co-continuous interlocking PDMS/PLA were calculated. Due to all co-continuous interlocking PDMS/PLA specimens’ density being higher than 1.0 g/cm^3^, the specific compressive strength and specific plateau stress slightly decreased compare with the compressive strength and plateau stress. With the strut diameter increasing, the specific compressive strength and specific plateau stress increased. Similarly, the PDMS/PLA-7 specimen still exhibited the highest specific compressive strength and the specific plateau stress in this study. This proves that the strut diameter of the PLA lattice as a skeleton is an important factor that can adjust the mechanical properties of the co-continuous interlocking PDMS/PLA. Figure 7 shows the compressive strength/plateau stress and corresponding density *ρ* of the co-continuous interlocking PDMS/PLA lattice composites. It was found that the compressive strength and the density are linearly correlated. Similarly, the plateau stress and the density are linearly correlated. The slope (208.16) of the compressive strength and density is slightly lower than that of the plateau stress and density (264.08), proving that the variation in density has a greater effect on the plateau stress of the co-continuous interlocking PDMS/PLA lattice composites.

### 3.3. Energy Absorption Behavior of Co-Continuous Interlocking PDMS/PLA Lattice Composites

**Figure 8:**
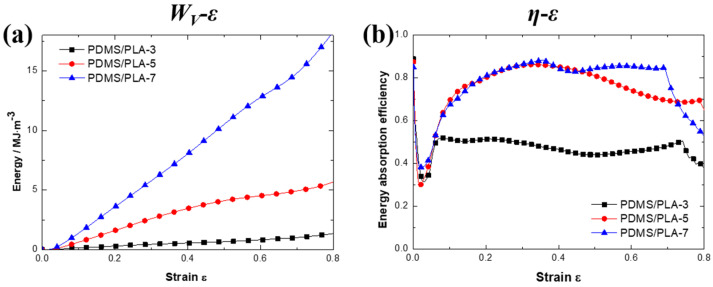
The *W_V_*-*ε* curves (**a**) and the energy absorption efficiency *η*-*ε* curves (**b**) of co-continuous interlocking PDMS/PLA lattice composites.

The energy absorption density is an important index used to characterize the energy absorption behavior of co-continuous interlocking PDMS/PLA lattice composites, reflecting the energy absorption capacity of materials or structures. The energy absorption density *W_V_* of co-continuous interlocking PDMS/PLA lattice composites can be calculated by Equation (1):

(1) $$W_{V}=\int_{0}^{\varepsilon }\sigma \left(\varepsilon \right)d\varepsilon$$

where *ε* and *σ* are the strain and corresponding stress, respectively. The *W_V_*-*ε* curves of the co-continuous interlocking PDMS/PLA lattice composites are plotted in Figure 8a. These curves are calculated using the average stress–strain curves for each condition. It is found that the enhancement of energy absorption density is significant with the increase of the strut diameter. For the PDMS/PLA-3 specimen, the *W_V_*-*ε* curves show a lower level of energy absorption during the whole compression process. The reason is that the PDMS/PLA-3 specimen exhibits a lower compressive stress level, resulting in a lower integral of stress on the strain. When the strut diameter increases to 5 mm, the energy absorption density is obviously improved. During the whole compression process, the energy absorption density of the PDMS/PLA-5 specimen is always higher than that of the PDMS/PLA-3 specimen. The higher stress level and the wider plateau region are both the reason for this result. The PDMS/PLA-7 specimen exhibits the highest level of energy absorption during the whole compression process. Even the energy absorption density of the PDMS/PLA-7 specimen is higher than the sum of the PDMS/PLA-3 specimen and the PDMS/PLA-5 specimen. It is observed that the effect of the strut diameter on energy absorption is significant for the co-continuous interlocking PDMS/PLA lattice composites. The PDMS/PLA-7 specimen has the largest strut diameter, resulting in the widest plateau region with largest plateau stress. These factors lead to the highest energy absorption density in this study.

The energy absorption efficiency reflects the ratio of the energy absorbed by a real material under any strain during compression to the energy absorbed by an ideal energy absorption material under the same strain. The energy absorption efficiency is another important index used to characterize the energy absorption behavior of co-continuous interlocking PDMS/PLA lattice composites. The energy absorption efficiency *η* of co-continuous interlocking PDMS/PLA lattice composites is calculated by Equation (2):

(2) $$\eta =\frac{\int_{0}^{\varepsilon }\sigma (\varepsilon )d\varepsilon }{\sigma_{max}\varepsilon }$$

where *σ*, *ε* and *σ_max_* are the stress, the strain and the maximum compressive stress in the strain range [0, *ε*], respectively. The *η*-*ε* curves of the co-continuous interlocking PDMS/PLA lattice composites are plotted in Figure 8b. These curves are calculated by the average stress–strain curves for each condition. 

As for the energy absorption efficiency, these specimens of the co-continuous interlocking PDMS/PLA lattice composites present an obvious difference. For the PDMS/PLA-3 specimen, the energy absorption efficiency rises to about 0.5 at the initial stage. Then, a wide and stable region appears. At this stage, the energy absorption efficiency is maintained at the level of 0.5. Finally, the energy absorption efficiency enters a stage of rapid decline. The *η*-*ε* curve of the PDMS/PLA-5 specimen presents a long and gentle rise. Then, it enters a relatively narrow stable region and reaches the maximum. Unfortunately, the maximum efficiency cannot be maintained for a long time with the strain increasing. After the efficiency stable region, the energy absorption efficiency decays slowly to around 0.7. On the whole, the energy absorption efficiency of the PDMS/PLA-5 specimen is higher than that of the PDMS/PLA-3 specimen. It is worth noting that the energy absorption efficiency of specimen PDMS/PLA-7 shows different characteristics. Except for a similar slow rise region initially, the PDMS/PLA-7 specimen exhibits superior energy absorption efficiency including the highest efficiency peak (>0.8) and a wider efficiency stable region. The last region is a rapid decay region. The energy absorption efficiency of specimen PDMS/PLA-7 is undoubtedly the highest. 

According to Equation (2), it is found that *σ_max_* has a direct impact on the energy absorption efficiency. For the PDMS/PLA-3 specimen, there is a stress peak on the compressive stress–strain curves. Meanwhile, the compressive strength is much higher than the plateau stress due to the stress drop. This makes *σ_max_* much higher than the stress at the subsequent strain. Therefore, the energy absorption efficiency of specimen PDMS/PLA-3 is relatively low. For the PDMS/PLA-5 specimen, the compressive strength and plateau stress are almost identical, meaning that *σ_max_* is close to the plateau stress at the subsequent strain. This improves the energy absorption efficiency of specimen PDMS/PLA-5. However, the decay stage comes early due to the narrow plateau region. For the PDMS/PLA-7 specimen, a wider plateau region makes *σ_max_* almost equal to the plateau stress within the plateau region. This contributes to obtaining a wider stable region and higher energy absorption efficiency. This result reflects that the strut diameter has an obvious impact on the energy absorption efficiency. 

**Figure 9:**
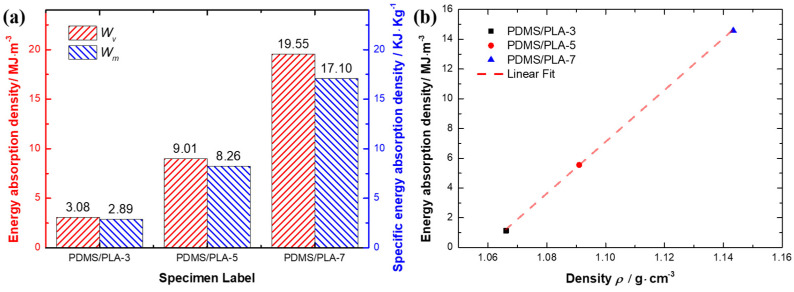
(**a**) The energy absorption density and the specific energy absorption density of co-continuous interlocking PDMS/PLA lattice composites. (**b**) The energy absorption density and corresponding density *ρ* of co-continuous interlocking PDMS/PLA lattice composites.

The energy absorption density of co-continuous interlocking PDMS/PLA lattice composites at the 0.6 strain is depicted in Figure 9a. At the same time, the effect of the specimen density on energy absorption is considered. Therefore, the energy absorption density *W_V_* at the 0.6 strain and the specific energy absorption density *W_m_* at the 0.6 strain of co-continuous interlocking PDMS/PLA lattice composites are comparatively analyzed. The specific energy absorption density *W_m_* is defined as *W_V_*/*ρ*. However, considering the effect of density, the PDMS/PLA-7 specimen still exhibits the highest specific energy absorption density. Figure 9b shows the energy absorption density and corresponding density *ρ* of co-continuous interlocking PDMS/PLA lattice composites. It is found that the energy absorption density and the density are still linearly correlated. The energy absorption density increases with the specimen density increasing. This result verifies that the variation of density can affect the energy absorption density of co-continuous interlocking PDMS/PLA lattice composites.

### 3.4. Effect of PLA Lattice Volume Fraction on Compressive Properties and Energy Absorption

**Figure 10:**
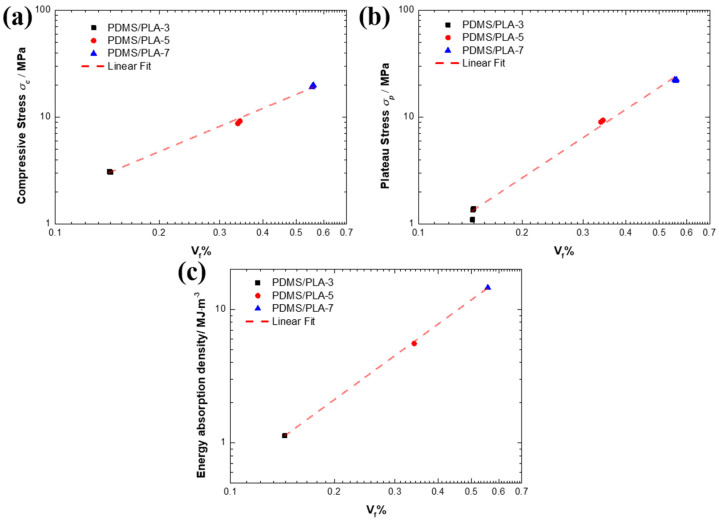
The relationships between (**a**) compressive strength, (**b**) plateau stress, (**c**) energy absorption density and PLA volume fraction *V_f_* of the co-continuous interlocking PDMS/PLA lattice composites.

As the co-continuous interlocking structure, the two phases, PLA and PDMS, play different roles. The PDMS fills the pores of the PLA lattice and wraps the PLA struts, playing a supporting role. Therefore, the PDMS as a filler prevents the negative effects resulting from the sudden rupture of PLA struts. Based on the aforementioned analysis, it is found that the PLA lattice acting as skeleton plays an important role in bearing the compressive load and energy absorption. In this study, changing the strut diameter actually alters the volume fraction of the PLA. With the strut diameter increasing, the volume of the PLA lattice increases. The relationships between the compressive strength, plateau stress, energy absorption density and the PLA volume fraction *V_f_* of the co-continuous interlocking PDMS/PLA lattice composites are plotted in the Figure 10. The compressive strength *σ_c_* and PLA volume fraction *V_f_* are linear in logarithmic coordinates (Figure 10a). With the PLA volume fraction increasing, the compressive strength increases. The slope and intercept are 1.349 and 1.614, respectively.

Similarly, the plateau stress *σ_p_* and PLA volume fraction *V_f_* show a similar linear relationship in the logarithmic coordinates (Figure 10b). The slope and intercept are 2.127 and 1.914, respectively. The effect of the PLA volume fraction increasing on the plateau stress is more sensitive, meaning that the increase in the plateau stress is higher than the increase in compressive strength with the same volume fraction variation. For the energy absorption density *W_m_* and PLA volume fraction *V_f_*, a linear relationship is presented with a slope of 1.881 and an intercept of 1.634 (Figure 10c). The energy absorption density increases with the PLA volume fraction increasing. Therefore, the relationships between the compressive strength, plateau stress, energy absorption density and PLA volume fraction of the co-continuous interlocking PDMS/PLA lattice composites are all linear in logarithmic coordinates.

The PDMS/PLA lattice composites were developed based on lattice structures. For practical applications of lattice structures in energy absorption, the PDMS/PLA lattice composites can also be utilized. For instance, the PDMS/PLA lattice composites have the potential to be used in protection devices such as the crush box of automotive bumper systems due to the lightweight and energy absorption characteristics. The practical application will be examined in the ongoing study. 

## 4. Conclusions

The conclusions are mainly summarized as follows:

- Co-continuous interlocking PDMS/PLA lattice composites were successfully manufactured using the FDM technique and infiltration method. The changes of structure parameters can be simultaneously realized using this fabrication method.
- The compressive properties of the co-continuous interlocking PDMS/PLA lattice composites can be adjusted in a narrow density range, simultaneously demonstrating a linear correlation characteristic. With the increase in the PLA strut diameter, the energy absorption density of the co-continuous interlocking PDMS/PLA lattice composites increases. Simultaneously, the energy absorption efficiency presents a higher efficiency peak and wider plateau region.
- The PLA lattice acting as skeleton plays an important role in bearing the compressive load and energy absorption. The relationships between the compressive strength, plateau stress, energy absorption density and the PLA volume fraction of co-continuous interlocking PDMS/PLA lattice composites are all linear in logarithmic coordinates. The effect of the PLA volume fraction increasing on the plateau stress is more sensitive than the compressive strength and energy absorption density.
- The PDMS/PLA lattice composites usually show superior compressive properties and energy absorption behavior compared with polymer lattice due to the introduction of a filler. Compared with metallic or ceramic lattice composites, they usually exhibit lower compressive strength and lightweight characteristics.

## Figures and Tables

**Table 1:** The specimen label, filler, strut diameter, mass and density of the PLA lattice structure and co-continuous interlocking PDMS/PLA lattice composites.

Specimen Label		Filler	*d*/mm	*m*/g	*ρ*/g·cm^−3^
1	PLA-3	/	3	7.151 ± 0.018	0.153 ± 0.000
2	PDMS/PLA-3	PDMS	3	49.743 ± 0.142	1.066 ± 0.003
3	PLA-5	/	5	17.358 ± 0.157	0.372 ± 0.003
4	PDMS/PLA-5	PDMS	5	50.902 ± 0.176	1.091 ± 0.004
5	PLA-7	/	7	29.750 ± 0.074	0.638 ± 0.002
6	PDMS/PLA-7	PDMS	7	53.346 ± 0.285	1.143 ± 0.006

## Data Availability

The raw data supporting the conclusions of this article will be made available by the authors upon request.
